# The roles of lifestyle factors and genetic risk in the association between night shift work and cholelithiasis: a prospective cohort study

**DOI:** 10.3389/fendo.2025.1573203

**Published:** 2025-05-28

**Authors:** Wangping He, Ningning Mi, Kecheng Jin, Boru Jin, Ruyang Zhong, Zhen Liu, Yanyan Lin, Hengwei Zhang, Ping Yue, Bin Xia, Qiangsheng He, Jinqiu Yuan, Wenbo Meng

**Affiliations:** ^1^ The First School of Clinical Medicine, Lanzhou University, Lanzhou, Gansu, China; ^2^ Department of General Surgery, The First Hospital of Lanzhou University, Lanzhou, Gansu, China; ^3^ Clinical Research Center, Big Data Center, The Seventh Affiliated Hospital, Sun Yat-sen University, Shenzhen, Guangdong, China

**Keywords:** night shift work, cholelithiasis, genetic risk, lifestyle factors, cohort study

## Abstract

**Background:**

Night shift work has been associated with adverse health outcomes. Whether night shift work is associated with cholelithiasis remains uncertain, and the roles of genetic risk and lifestyle factors in cholelithiasis risk are unclear.

**Methods:**

We conducted a prospective analysis of 219,810 UK Biobank participants. Cox proportional hazards models were used to estimate the association between night shift work and incident cholelithiasis. Polygenic risk score analyses and causal mediation analyses were performed to investigate the roles of the genetic risk and lifestyle factors in cholelithiasis risk.

**Results:**

Compared with day workers, the HR and 95% CI of cholelithiasis was 1.09 (1.01-1.17) for individuals with rarely/some night shifts and 1.18 (1.04-1.35) for those with usual/permanent night shifts. Additionally, those with a higher frequency of night shifts and a longer length of each night shift were associated with an increased risk of cholelithiasis. Notably, individuals with usual/permanent night shifts and high genetic risk exhibited the highest risk of cholelithiasis (HR: 1.48, 95% CI: 1.21-1.81). Mediation analysis indicated that a substantial portion (24.64%) of the association was mediated by BMI, followed by unhealthy alcohol intake (4.50%) and sedentary time (1.82%).

**Conclusions:**

Night shift work is associated with an increased risk of cholelithiasis, with this relationship being largely mediated by lifestyle factors. Reducing the frequency and length of night shifts may help mitigate the incidence of cholelithiasis among night shift workers, particularly for those with heightened genetic susceptibility.

## Introduction

Cholelithiasis is characterized by the presence of gallstones in the gallbladder or bile duct, and it affects 10–20% of the global adult population and contributes significantly to the healthcare burden (1). Identifying and controlling emerging risk factors is crucial for mitigating the healthcare impact of this condition. Approximately 15-20% of the working population in Europe and the United States engages in night shift work (2). Although necessary in many sectors, previous studies have linked night shift work to elevated risks of various health conditions, including cardiovascular disease, metabolic disorders, and certain cancers (3–6), with the impact potentially intensifying with prolonged exposure to night shifts. While the mechanisms linking night shift work to cardiovascular disease have been extensively studied (7), relatively few investigations have examined its association with digestive diseases. Existing literature suggests that night shift work correlates positively with conditions such as peptic ulcers, constipation, indigestion (8), gastritis (9), gastroesophageal reflux disease (GERD) (10), irritable bowel syndrome (IBS) (11), nonalcoholic fatty liver disease (NAFLD) (12), and cancers of the biliary tract (13) and colorectum (14). However, the potential risk of cholelithiasis associated with night shift work, particularly with prolonged and frequent exposure, remains unclear.

Notably, unhealthy lifestyle factors, such as higher body mass index (BMI), unhealthy diet, smoking, alcohol consumption, sleep disorders, sedentary behavior, and lack of physical activities, are known to increase the risk of these digestive diseases that are significantly associated with night shift work (15–19). Moreover, many risk factors for chronic diseases can influence lifestyle behaviors, ultimately contributing to the onset of these diseases (20–23). This perspective applies equally to cholelithiasis: night shift work—a unique occupational schedule—has a significant negative impact on some lifestyle factors (24–26). Consequently, night shift work may increase the risk of cholelithiasis both directly and indirectly by altering lifestyle behaviors. In this context, lifestyle factors likely serve as crucial mediators in the relationship between night shift work and cholelithiasis risk. Specifically, night shift work may increase cholelithiasis risk through the pathway: night shift work → adverse lifestyle behaviors → cholelithiasis. Treating lifestyle factors merely as confounders and adjusting for them in statistical models could lead to overadjustment bias (27), potentially masking the true association between night shift work and cholelithiasis. Therefore, it is essential to further investigate the potential mediating role of lifestyle factors in the relationship between night shift work and cholelithiasis to gain a more comprehensive understanding of how night shift work influences disease risk.

In addition to environmental factors, genetic predisposition plays a significant role in the development of cholelithiasis (28). Large-scale genome-wide association studies (GWAS) have identified several independent genetic loci linked to an increased risk of cholelithiasis in European populations (29, 30). These risk-associated variants have contributed to the development of polygenic risk scores (PRS), which effectively identify individuals at high risk for developing cholelithiasis (31, 32). Genetic predisposition influences disease susceptibility, which may modify the relationship between environmental factors, such as night shift work, and the development of chronic disease (33). However, to date, no study has explored whether the association between night shift work and cholelithiasis varies by genetic predisposition, quantified by PRS. Therefore, the exploration of the combined effects of night shift work and genetic risk will deepen our understanding of how genetic susceptibility may amplify the impact of environmental risk factors. This exploration may also suggest that the impact of night shift work varies depending on an individual’s genetic background. These investigations could facilitate the development of personalized, precision prevention strategies for cholelithiasis, emphasizing the need for primary prevention approaches tailored to the genetic susceptibility of night shift workers.

To address these gaps in knowledge, we utilized data from the UK Biobank to investigate the relationships between exposure to night shift work and cholelithiasis risk. Additionally, we integrated information on in-depth lifetime employment to examine the impact of the length and frequency of night shifts on cholelithiasis risk. For gene-environment interactions, we constructed a specific PRS for cholelithiasis to investigate the combined effects of night shift work and genetic variations on the cholelithiasis risk. Given that night shift work negatively impacts health through suboptimal health lifestyles (34) and certain lifestyle factors are likely to play a mediating role, guided by the American Heart Association’s Life’s Essential 8 (35), prior evidence (36), and established associations with night shift work or cholelithiasis, we explored mediation effects of seven lifestyle factors: BMI, smoking status, alcohol intake, sleep duration, physical activity, sedentary time, and dietary characteristics.

## Materials and methods

### Study population

The study population was drawn from the UK Biobank, a large-scale prospective cohort study that recruited over 500,000 individuals aged 37 to 73 years from 22 assessment centers across England, Scotland, and Wales between 2006 and 2010 (37). At baseline, participants provided electronic consent and completed a touch-screen questionnaire along with a face-to-face interview. Trained staff collected various measurements, including height and weight. Participants also provided detailed information on their lifestyle, medical conditions, work hours, and demographic background. Health professionals inquired about medical history, health status, and medication use. Further details have been extensively documented in prior studies (38, 39).

For our analyses, we focused on participants who were in paid employment or self-employed (n = 286,248). After excluding participants with a history of cholelithiasis or cholecystectomy at baseline and missing lifestyle information and covariates, 219,810 participants were included in the primary analysis. Among them, 165,282 subjects were included in the subsequent gene-environment interaction analysis. Of those, 62,558 subjects have complete lifetime employment information from the follow-up in 2015 through online questionnaires (**Supplementary Figure S1**).

Within this prospective population-based cohort study, the duration of follow-up was calculated from the baseline (2006-2010) to the first diagnosis of cholelithiasis, death, or the date of last follow-up (31 MAY 2022), whichever came first.

### Exposure assessment

Shift work was defined as a work schedule outside normal daytime working hours of 9:00 AM to 5:00 PM, which means working afternoons, evenings or nights or rotating through these shifts. Participants who indicated paid employment or self-employment were then asked “Does your work involve shift work?”. If yes, participants were further asked whether their main job involved night shifts, defined as “…a work schedule that involves working through the normal sleeping hours (e.g., from 12:00 a.m. to 6:00 a.m.).” Response options for both questions included “never/rarely,” “sometimes,” “usually,” or “always,” with additional options for “prefer not to answer” and “do not know” (40). Based on this information, participants’ current night shift work status was categorized as “day workers” “shift, but rarely/some night shifts” and “usual/permanent night shifts”.

Additionally, 62,558 participants provided detailed lifetime employment information by completing an online follow-up questionnaire in 2015. This questionnaire was sent to approximately 330,000 UK Biobank participants with known email addresses, regardless of their baseline employment status or shift work history (41). In this follow-up, participants reported each job ever worked, the number of years in each job, and the number of night shifts per month for each job. The detailed assessment method has been described in previous studies (3, 40). Based on the above information, we calculated the average frequency (number/per month) of night shifts and average length (hours) of each night shift for each participant.

### Outcome ascertainment

The primary outcome of interest was cholelithiasis. The diagnosis of cholelithiasis was based on medical history and linkage to the hospital statistics from England, Scotland and Wales, which was defined according to the International Classification of Diseases (10th revision, ICD-10), as coded K80.

### Assessment of polygenic risk score to cholelithiasis

Genotyping in the UK Biobank was performed on two arrays, UK BiLEVE and UK Biobank Axiom. Genotyping, quality control, and imputation procedures have been detailed previously (42). The polygenic risk score (PRS) was computed using the cholelithiasis-associated single nucleotide polymorphisms (SNPs) identified in our previous study (43). Detailed information about 13 independent SNPs is provided in **Supplementary Table S1**, and the full process of PRS development and validation is described in our previous work (43). In short, the number of alleles (0, 1, or 2) for each individual was multiplied by the effect size of SNPs associated with cholelithiasis, and then summed to derive PRS for all individuals in the UK Biobank (32). The PRS was formulated as: PRS = *β*_1_ × SNP_1_ + *β*_2_ × SNP_2_ + … + *β*_n_ × SNP_n_ (*β*_n_ represents the effect size of the n-th SNP, and SNP_n_ indicates the genotype value for the n-th SNP, indicating the number of risk alleles (0, 1, or 2) present in the individual), with a higher score indicating greater genetic susceptibility to cholelithiasis. We then determined whether participants were at high or low genetic risk for cholelithiasis based on the median value of PRS.

### Assessment of covariates and lifestyle factors

Age, gender, race, UK Biobank assessment centers, index of multiple deprivation, education, health rating, long-standing illness, multivitamin use and mineral use were considered as potential covariates. Index of multiple deprivation (IMD) reflecting socioeconomic status and different research centers acquired directly from the UK Biobank. Other sociodemographic characteristics (age, gender, race, education) were collected through questionnaires or verbal interviews at baseline and health status (health rating, long-standing illness, multivitamin use and mineral use) was collected through the self-reported medical history or hospitalization records.

Lifestyle factors included status of BMI, smoking status, alcohol intake, sleep duration, physical activity, sedentary time, and dietary characteristics (**Supplementary Table S2**). Weight and height were measured at baseline during the initial assessment center visit. Body mass index (BMI) was calculated as weight divided by height squared (kg/m^2^) at the initial assessment center visit. Smoking status, alcohol intake, sleep duration was assessed by the touch-screen questionnaire. Physical activity was assessed using the validated short International Physical Activity Questionnaire (IPAQ) (44), which measures time spent on walking, and moderate and vigorous activities, which were also assessed by the touchscreen questionnaire. We used TV time as a measure of sedentary time (45). Dietary characteristics were assessed based on the intake of 5 dietary components (fruits, vegetables, fish, red meat, and processed meat) following the American Heart Association Guideline (46).

### Statistical analysis

Baseline characteristics were presented as mean (standard deviation, SD) for continuous variables, and as a number (percentage) for categorical variables. The person-years of follow-up were calculated from the assessment date for each participant until the first diagnosis of cholelithiasis, death, or the date of last follow-up (31 MAY 2022), whichever came first.

We employed Cox proportional hazards models to estimate the hazard ratios (HRs) and 95% confidence intervals (CIs) for the association between current night shift work, average lifetime night shift frequency and average length of each night shift during night shift periods and incident cholelithiasis. Proportional hazards assumptions were verified for all Cox models using Schoenfeld residual tests, with no evidence of violation.

Three models with increasing adjustment were utilized to control potential confounders. Model 1 was stratified by age, sex, and UK Biobank assessment centers. Model 2 was further adjusted for model 1 plus race, index of multiple deprivation and education level. In Model 3, we further adjusted for health rating, long-standing illness, multivitamin use and mineral use.

We estimated cumulative incidence curves by genetic risk for cholelithiasis. The study participants were then divided into six groups based on genetic risk (high genetic risk, low genetic risk) and night shift work status (“day workers” “shift, but rarely/some night shifts” and “usual/permanent night shifts”). Using day workers with low genetic risk as the reference group, the combined effect of genetic risk and night shift work on the incidence of cholelithiasis was evaluated by directly comparing the hazard ratios (HRs) of these subgroups using Cox models.

We examined seven potential lifestyle mediators of the association between night shift work and cholelithiasis: BMI, smoking status, alcohol intake, sleep duration, physical activity, sedentary time, and dietary characteristics. All potential lifestyle mediators were selected due to their association with night shift work and/or cholelithiasis. First, these potential lifestyle mediators were adjusted in the Cox models to examine whether, and to what extent, the associations between night shift work and cholelithiasis were attenuated as an exploration of mediation. Subsequently, a formal mediation analysis based on a counterfactual framework was then performed to assess the mediating roles of lifestyle factors in this association. The counterfactual framework formally defines both direct (non-mediated) and indirect (mediated) effects, and is more robust to the various limitations of traditional adjustment-based mediation analysis, such as mediator–outcome confounding affected by exposure (47). The natural indirect effect (NIE), natural direct effect (NDE), and total effect (TE) were estimated by combining mediation and outcome models that adjusted for covariates in Model 3. Mediation proportion was calculated as NIE/TE. Nonparametric bootstrap resampling was used to compute the 95%CIs of mediation proportions.

We estimated the association between night shift work and each lifestyle factor using the multivariate-adjusted linear or logistic regression models. The associations between lifestyle factors and the risk of cholelithiasis was investigated using multivariate-adjusted Cox regression models (**Supplementary Tables S3**, **S4**).

To examine the robustness of the results, several sensitivity analyses were conducted. First, we excluded incident cases occurring within the first year to reduce the possibility of reverse causation, enhance temporal considerations, and allow a time window for the development of cholelithiasis. Additionally, we tested whether chronotype modified the association between night shift work and the risk of cholelithiasis by adjusting for the chronotype category in the models. Lastly, we analyzed the impact of night shift work on cholelithiasis using Fine-Gray methods accounting for death as a competing risk, to assess the robustness of our findings. In the latter analysis, current night shift work status was categorized as “day workers” and “night shift workers” instead of three classifications applied in the main analysis. All statistical analyses were performed using R software (version 4.3.2) and Python (version 3.7.13), and a two-sided p-value < 0.05 was considered statistically significant.

## Results

### Baseline characteristics of participants

**Table 1** presents the baseline characteristics of the study population according to current night shift status. In general, among the 219,810 participants, night shift workers were more likely to be younger, male, nonwhite, less educated and more physically active and have higher BMI, unhealthy lifestyles and lower socioeconomic status. These workers also exhibited higher prevalences of long-standing illnesses and self-reported poorer overall health. The baseline characteristics of participants according to average monthly frequency of night shifts and average length of each night shift are presented in **Supplementary Tables S5**, **S6**. These characteristics were further accentuated by the increase in the frequency and duration of night shifts.

**Table 1:** UK Biobank participants’ characteristics by current night shift work exposure (*n *= 219,810).

Characteristics	Day workers (N=184817)	Shift, but rarely/some night shifts (N=27518)	Usual/permanent night shifts (N=7475)	Overall (N=219810)
**Mean (SD) Age, years**	52.7 (7.09)	51.7 (7.00)	51.0 (6.77)	52.5 (7.08)
Gender, N (%)				
Female	94079 (50.9)	12180 (44.3)	2597 (34.7)	108856 (49.5)
Male	90738 (49.1)	15338 (55.7)	4878 (65.3)	110954 (50.5)
**Mean (SD) Index of multiple deprivation**	15.6 (12.5)	19.7 (14.9)	21.8 (15.7)	16.3 (13.0)
**Mean (SD) body mass index, kg/m^2^**	26.9 (4.52)	27.7 (4.80)	28.3 (4.78)	27.1 (4.58)
Health rating, N (%)				
Excellent	38871 (21.0)	4337 (15.8)	1113 (14.9)	44321 (20.2)
Good	111484 (60.3)	16331 (59.3)	4324 (57.8)	132139 (60.1)
Poor	3438 (1.9)	760 (2.8)	242 (3.2)	4440 (2.0)
**Long-standing illness, N (%)**	43420 (23.5)	7250 (26.3)	1951 (26.1)	52621 (23.9)
Education level, N (%)				
A levels/AS levels or equivalent	24058 (13.0)	3391 (12.3)	841 (11.3)	28290 (12.9)
College or University degree	81664 (44.2)	7502 (27.3)	1267 (16.9)	90433 (41.1)
CSEs or equivalent	10410 (5.6)	2569 (9.3)	925 (12.4)	13904 (6.3)
NVQ or HND or HNC or equivalent	10956 (5.9)	2578 (9.4)	827 (11.1)	14361 (6.5)
O levels/GCSEs or equivalent	37304 (20.2)	6712 (24.4)	2074 (27.7)	46090 (21.0)
Other	6939 (3.8)	1579 (5.7)	545 (7.3)	9063 (4.1)
**White, N (%)**	175902 (95.2)	24850 (90.3)	6553 (87.7)	207305 (94.3)
**Multivitamin use, N (%)**	26105 (14.1)	3924 (14.3)	926 (12.4)	30955 (14.1)
**Intake of mineral supplements, N (%)**	41155 (22.3)	6333 (23.0)	1628 (21.8)	49116 (22.3)
Dietary characteristics, N (%)				
Unhealthy diet	93734 (50.7)	14471 (52.6)	4190 (56.1)	112395 (51.1)
Healthy diet	91083 (49.3)	13047 (47.4)	3285 (43.9)	107415 (48.9)
Smoking status, N (%)				
Unhealthy smoking	17394 (9.4)	3895 (14.2)	1228 (16.4)	22517 (10.2)
Healthy smoking	167423 (90.6)	23623 (85.8)	6247 (83.6)	197293 (89.8)
Alcohol intake, N (%)				
Healthy alcohol intake	175203 (94.8)	25495 (92.6)	6794 (90.9)	207492 (94.4)
Unhealthy alcohol intake	9614 (5.2)	2023 (7.4)	681 (9.1)	12318 (5.6)
Physical activity, N (%)				
Healthy physical activity	146714 (79.4)	23411 (85.1)	6513 (87.1)	176638 (80.4)
Unhealthy physical activity	38103 (20.6)	4107 (14.9)	962 (12.9)	43172 (19.6)
Sleep duration, N (%)				
Healthy sleep duration	139745 (75.6)	19007 (69.1)	4530 (60.6)	163282 (74.3)
Unhealthy sleep duration	45072 (24.4)	8511 (30.9)	2945 (39.4)	56528 (25.7)
Watch TV, N (%)				
Healthy TV viewing time	153273 (82.9)	21508 (78.2)	5426 (72.6)	180207 (82.0)
Unhealthy TV viewing time	31544 (17.1)	6010 (21.8)	2049 (27.4)	39603 (18.0)

A levels/AS levels, advanced levels/advanced subsidiary levels; O levels/GCSE, ordinary levels/general certificate of secondary education; CSE, certificate of secondary education; NVQ, national vocational qualification; HND, higher national diploma; HNC, higher national certificate;

### Associations of current night shift work with incident cholelithiasis

During a median follow-up of 13.76 years, we documented 6450 incidents of cholelithiasis. We first evaluated the association of current night shift work status and the risk of cholelithiasis (2). Compared with day workers, increasing categories of night shifts were associated with a higher risk of incident cholelithiasis in age-, sex-, and UK Biobank assessment centers-stratified model 1 (P for trend <0.001), and rotating shifts with usual or permanent night shifts had the highest risk (HR 1.36, 95% CI [1.20-1.55]). This association remained significant after adjusting for race, index of multiple deprivation and education level in Model 2. In the final model further adjusted for health rating, long-standing illness, multivitamin use and mineral use, a significant positive trend still existed between current night shift and risk of cholelithiasis (P for trend 0.001). In sensitivity analyses, when we excluded cholelithiasis cases within the first year or further adjusted for chronotype category in the models (**Supplementary Tables S7**, **S8**), the results did not substantially differ from those observed in our aforementioned analyses. In addition, the application of competing risk models demonstrated that night shift work is still an independent risk factor for cholelithiasis (**Supplementary Table S9**).

Lifestyle factors were selected as potential mediators and were initially adjusted in the Cox models as an exploration of mediation (**Table 2**). The associations between night shift work and cholelithiasis were attenuated further after adjusting for potential lifestyle mediators. The strongest attenuation was observed after adjusting for BMI (the HR and 95% CI before adjustment: 1.09 [1.01,1.17] in rarely/some night shifts and1.18 [1.04,1.35] in rarely/some night shifts; after adjustment: 1.05 [0.98,1.13] in rarely/some night shifts and1.12 [0.99,1.28] in rarely/some night shifts).

**Table 2:** Associations between current night shift work and risk of cholelithiasis (*n* =219,810).

Variables	HR (95% CI) by current work schedule			P for trend
	Day workers	Shift, but rarely/some night shifts	Usual/permanent night shifts	
Cases	5292	899	259	
Person-years	2485075.9	367718.5	100067.0	
Incidence rate*	21.30	24.45	25.88	
Model 1	1.00 (ref)	1.21 (1.13-1.30)	1.36 (1.20-1.55)	<0.001
Model 2	1.00 (ref)	1.12 (1.04-1.21)	1.22 (1.07-1.39)	<0.001
Model 3	1.00 (ref)	1.09 (1.01-1.17)	1.18 (1.04-1.35)	0.001
+ dietary characteristics	1.00 (ref)	1.09 (1.01-1.17)	1.18 (1.04-1.35)	0.001
+ smoking status	1.00 (ref)	1.08 (1.01-1.17)	1.18 (1.04-1.34)	0.002
+ alcohol intake	1.00 (ref)	1.09 (1.01-1.17)	1.18 (1.04-1.34)	0.001
+ physical activity	1.00 (ref)	1.10 (1.02-1.19)	1.21 (1.06-1.37)	<0.001
+ sleep duration	1.00 (ref)	1.09 (1.01-1.17)	1.18 (1.04-1.34)	0.002
+ TV viewing^†^	1.00 (ref)	1.08 (1.01-1.17)	1.17 (1.03-1.33)	0.002
+ BMI	1.00 (ref)	1.05 (0.98-1.13)	1.12 (0.99-1.28)	0.032

ref, reference; BMI, body mass index.

*Per 10,000 person-years.

^†^TV viewing was used to measure sedentary time.

Model 1 stratified by age, sex and UK Biobank assessment centers; Model 2 further adjusted for race, index of multiple deprivation and education level; Model 3 adjusted for terms in Model 2 and health rating, long-standing illness, multivitamin use and mineral use.

### Mediation analyses

Mediation analyses are summarized in **Table 3**. Lifestyle factors associated with both night shift work and outcomes were selected for mediation analysis (**Supplementary Tables S3**, **S4**). The primary mediators identified were unhealthy alcohol intake (4.5%), unhealthy TV viewing or sedentary time (1.8%), and BMI (24.2%). Detailed mediation charts depicting the natural indirect effect (NIE) and natural direct effect (NDE) of each lifestyle factor are provided in **Supplementary Figure S2**.

**Table 3:** Summary of mediation analyses.

Characteristics	Association with night shift work ^a^	Incident cholelithiasis	
		Association with outcome ^b^	% Mediated (95% CI)
**Unhealthy dietary**	–	+	NA
**Current smoking**	+	0	NA
**Unhealthy alcohol intake**	+	+	4.50(0.15-8.81)
**Low physical activity**	–	+	NA
**Unhealthy sleep duration**	+	0	NA
**Unhealthy TV viewing or sedentary time**	+	+	1.82(0.45-3.81)
**BMI**	+	+	24.64(5.79-35.53)

+, positive association; -, negative association; 0, no significant association; BMI, body mass index; TV, television; NA, not available.

a Summarized from **Supplementary Table S3**: multiple linear or logistic regression models with the potential mediator as the dependent variable and night shift work as the independent variable. Stratified by age, sex, and UK Biobank assessment centers and additionally adjusted for each other and for race, index of multiple deprivation, education level, health rating, long-standing illness, multivitamin use and mineral use.

b Summarized from **Supplementary Table S4**: Cox regression models with potential mediators as independent variables. Stratified by age, sex, and UK Biobank assessment centers and additionally adjusted for each other and for race, index of multiple deprivation, education level, health rating, long-standing illness, multivitamin use and mineral use.

### Associations of lifetime night shift work with incident cholelithiasis

We then examined the associations between lifetime night shift work (frequency and length) and the risk of cholelithiasis among 62,558 participants, with 1,578 incident cases. In fully adjusted models, increased night shift frequency was associated with a higher risk of cholelithiasis (**Table 4**). Compared with day workers, the HR (95% CI) of cholelithiasis was 1.18 (1.02, 1.37) in <8 night shifts/month and 1.26 (1.08, 1.46) in ≥8 night shifts/month. Additionally, we observed that night shift workers who undertook>12 hours per shift had the highest cholelithiasis risk (HR 1.25 [95% CI 1.06, 1.48]) (**Table 5**).

**Table 4:** Associations between average lifetime night shift frequency and risk of cholelithiasis (*n* = 62,558).

Variables	HR (95% CI) by average lifetime night shift frequency			*P* for trend
	None	<8/month	≥8/month	
Cases	1122	232	224	
Person-years	645107.98	111648.07	92601.68	
Incidence rate*	17.39	20.78	24.19	
Model 1	1.00 (ref)	1.26 (1.10-1.46)	1.48 (1.28-1.71)	<0.001
Model 2	1.00 (ref)	1.20 (1.04-1.39)	1.31 (1.13-1.53)	<0.001
Model 3	1.00 (ref)	1.18 (1.02-1.37)	1.26 (1.08-1.46)	0.001

ref= reference.

*Per 10,000 person-years.

Model 1 stratified for age, sex and UK Biobank assessment centers; Model 2 further adjusted for race, index of multiple deprivation and education level; Model 3 adjusted for terms in Model 2 and health rating, long-standing illness, multivitamin use and mineral use.

**Table 5:** Associations between average length of each night shift and risk of cholelithiasis (*n* = 62,558).

Variables	HR (95% CI) by average length of each night shift during night shift periods				*P* for trend
	None	≤8h	8-12h	≥12h	
Cases	1122	166	126	164	
Person-years	645107.98	76709.80	56175.72	71364.23	
Incidence rate*	17.39	21.64	22.43	22.98	
Model 1	1.00 (ref)	1.40 (1.18-1.65)	1.29 (1.07-1.55)	1.38 (1.17-1.63)	<0.001
Model 2	1.00 (ref)	1.27 (1.07-1.51)	1.18 (0.97-1.42)	1.30 (1.09-1.53)	<0.001
Model 3	1.00 (ref)	1.23 (1.04-1.46)	1.16 (0.95-1.40)	1.25 (1.06-1.48)	0.002

ref= reference.

*Per 10,000 person-years.

Model 1 stratified for age, sex and UK Biobank assessment centers; Model 2 further adjusted for race, index of multiple deprivation and education level; Model 3 adjusted for terms in Model 2 and health rating, long-standing illness, multivitamin use and mineral use.

### Combined effects of night shift work and genetic variations on the cholelithiasis risk

**Figure 1:**
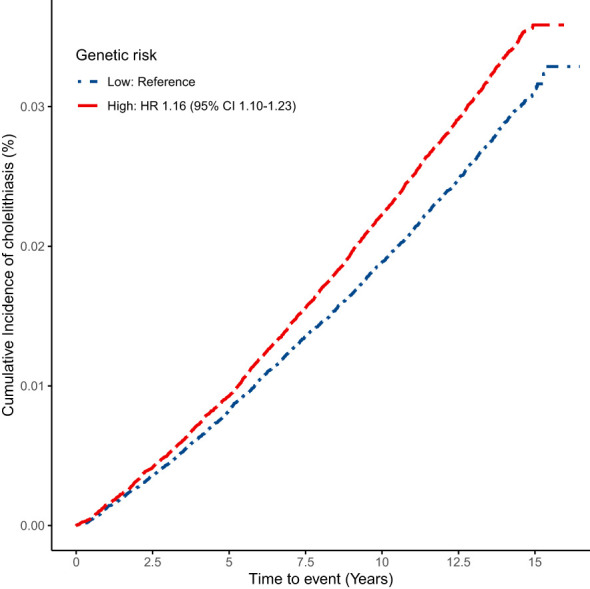
Cumulative incidence by genetic risk for cholelithiasis.

**Figure 2:**
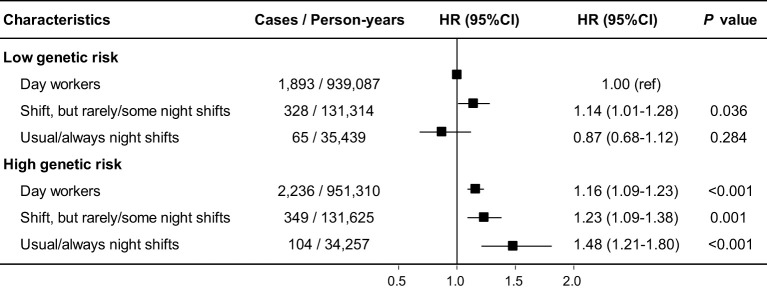
Joint effects of genetic risk with current night shift work on cholelithiasis risk. Hazard ratios were stratified by age, sex and UK Biobank assessment centers and additionally adjusted for race, index of multiple deprivation, education level, health rating, long-standing illness, multivitamin use, mineral use and the first 10 principal components of ancestry.

The curve of cumulative incidence by genetic risk for cholelithiasis showed that genetic risk was significantly associated with cholelithiasis (**Figure 1**). As expected, participants with high genetic risk exhibited a significantly higher risk for cholelithiasis compared to those with a low genetic risk (HR 1.16, 95% CI [1.10, 1.23]). Furthermore, we observed significant joint effects between genetic risk and current night shift work on the cholelithiasis risk (**Figure 2**). Compared with day workers who had lower genetic risk, individuals with usual/permanent night shifts and high genetic risk had the highest risk of cholelithiasis (HR 1.48, 95% CI [1.21, 1.80]).

## Discussion

In this study, we identified a significant association between night shift work and an increased risk of cholelithiasis. The risk showed a progressive increase from day work to irregular night shifts and regular night shifts. Joint analysis revealed that participants with usual/permanent night shift work and high genetic risk exhibited the highest risk of incident cholelithiasis compared to day workers who had lower genetic risk. Additionally, longer night shift durations and greater night shift frequencies were associated with an elevated risk of cholelithiasis. From a lifestyle perspective, BMI, alcohol intake, and sedentary time could partially mediate the association between night shift work and cholelithiasis, with BMI demonstrating the most pronounced mediating effect. These findings shed light on potential targets for interventions to reduce the subsequent risk of cholelithiasis in population with higher genetic risk.

To the best of our knowledge, this is the first prospective cohort study to investigate the relationships between night shift work and the risk of incident cholelithiasis. While previous research has extensively studied the association between night shift work and cardiovascular diseases (48, 49), cancers (50, 51), and metabolic diseases (5), its adverse effects on digestive health have received limited attention. However, some studies have reported associations between night shift work and conditions such as gastritis (9), gastroesophageal reflux disease (GERD) (10), irritable bowel syndrome (IBS) (11), nonalcoholic fatty liver disease (NAFLD) (12), and cancers (13, 14). To some extent, our results may be partly supported by those findings. For example, Lee et al. reported that night shift workers experienced more frequent gastrointestinal symptoms. They also found that night shift workers had a higher risk of gastritis compared to day workers (9). Similarly, Huang et al. found that the increased risk of NAFLD among individuals with night shift work was partly attributable to elevated BMI (12). Moreover, BMI is a well-established risk factor for cholelithiasis (32). In this study, we observed that the night shift was a significant and novel risk factor for cholelithiasis. These findings not only enhance our understanding of the digestive health impacts of night shifts but also provide crucial evidence for developing primary prevention strategies for cholelithiasis.

Furthermore, we noted that a higher frequency of night shifts and a longer length of each night shift were important determinants of increased cholelithiasis risk. Similar findings have been reported in relation to NAFLD, where higher frequency and longer hours of night shift work were associated with increased risk (12). These results provide compelling evidence of the detrimental relationships between night shift work and digestive health, suggesting that reducing night shift frequency and duration may be effective in preventing digestive diseases among night shift workers.

The mechanism linking night shift work and cholelithiasis remains unclear, but there are several possible explanations. First, circadian disruption, light exposure at night, and sleep deprivation are characteristic features of night shift work (6). Evidence from animal studies has suggested that disordered circadian rhythm and abnormal cholesterol metabolism in mice promote gallstone formation (52). Additionally, unhealthy sleep traits may increase the risk of gallbladder disease (53). Exposure to light at night suppresses melatonin secretion and increases proinflammatory reactive oxygen species (6). Inflammatory changes in gall bladder mucosa and reduced gall bladder motility are considered as risk factors for cholelithiasis (54). Consequently, Sreedevi et al. proposed melatonin as a potential treatment for treat gallstones, targeting both reactive oxygen species and gallbladder hypomotility (55). Second, the expression of genes that encode key enzymes in hepatic bile acid and cholesterol metabolism, such as *Hmgcr* and *Cyp7a1*, is regulated by circadian rhythm-related transcription factors (56). Thus, the disturbance of circadian rhythm caused by night work can affect cholesterol metabolism and ultimately lead to the development of cholelithiasis. Moreover, the composition of the gut microbiota also shows circadian fluctuations (57), which when disturbed by night work may contribute to the development of cholelithiasis. These findings suggest that the disturbance of intestinal microbiota among night shift workers may be one of the reasons for the increased incidence of cholelithiasis. Finally, the circadian disruption can disturb diurnal rhythms in vagal afferents (58). The hepatic branch of the vagus nerve plays a critical role in biliary function, and damage to this branch can lead to impaired gallbladder contraction, the accumulation of bile salts, cholestasis, and eventually the formation of gallstones (59). Therefore, the high incidence of cholelithiasis among night shift workers may be attributed to abnormal vagal afferent rhythm.

Night shift work is often associated with unhealthy lifestyle behaviors, as it is frequently linked to factors such as low income, poor environmental conditions, and elevated stress levels, which may lead to unhealthy lifestyles (60). Therefore, in order to examine the pathways through which night shift work may contribute to cholelithiasis, we analyzed the mediating role of various lifestyle factors. After adjusting for lifestyle factors, the association between night shift work and cholelithiasis was partially attenuated. Mediation analysis revealed that lifestyle factors such as unhealthy alcohol intake, increased BMI, and sedentary behavior partially explained the relationship between night shift work and cholelithiasis risk. The impact of night shift work on lifestyle is primarily driven by its disruption of the body’s normal circadian rhythm. First, the fatigue and hormonal imbalances caused by night shift work can lead to increased BMI and obesity (61), which not only increases the cholesterol saturation in bile but also slows down gallbladder emptying (62), making obesity one of the most significant risk factors for cholelithiasis. Additionally, circadian rhythm disruption is often associated with increased sedentary behavior and poor dietary habits, which can promote insulin resistance and dyslipidemia (61), further indirectly increasing the likelihood of cholesterol stone formation. A long-term lack of regular meals can also affect normal gallbladder emptying: normal eating is a stimulus for gallbladder emptying, but night shift workers, due to disrupted meal timing or decreased appetite, may have their gallbladders in a constantly filled state, leading to bile stasis and an increased risk of stone formation (63). Finally, changes in alcohol consumption habits are another factor of concern. Moderate alcohol consumption can stimulate gallbladder contraction and increase high-density lipoprotein levels, which is thought to have a protective effect on gallstones (64). However, night shift workers may experience disrupted drinking patterns due to changes in social rhythms and increased work-related stress, and excessive alcohol consumption may exacerbate metabolic burdens (65). Unhealthy lifestyle factors are interwoven and, together with circadian rhythm disruption, create a fertile ground for the occurrence of cholelithiasis. In this context, these findings may contribute to a better understanding of the underlying mechanisms between night shift work and cholelithiasis, suggesting that while promoting healthy lifestyles may mitigate some adverse effects of night shift work, additional protective measures are necessary.

Cholelithiasis is a chronic condition influenced by the long-term interplay of genetic and environmental factors (54). Polygenic risk scores (PRS) that incorporate multiple single nucleotide polymorphisms (SNPs) provide a more comprehensive prediction of individual genetic risk for cholelithiasis compared to a single SNP, which only partially explains cholelithiasis susceptibility (30, 31). In this study, the PRS demonstrated robust utility in stratifying cholelithiasis risk, with high genetic risk individuals exhibiting a 16% increased hazard compared to low-risk counterparts (HR 1.16, 95% CI 1.11-1.23), suggesting that the PRS could effectively stratifies cholelithiasis risk. To our knowledge, no previous study has comprehensively examined the joint associations of PRS and night shift work in cholelithiasis risk. Consistent with our hypothesis, individuals with high genetic risk who engaged in regular or permanent night shift work exhibited the highest risk of developing cholelithiasis. Several biological pathways might explain the interaction between night shift work and genetic risk in relation to cholelithiasis. First, Fairfield et al. found that the PRS for cholelithiasis was strongly associated with increased BMI (31). Additionally, previous studies have confirmed that night shift work plays a significant role in increasing BMI (66, 67). Elevated BMI contributes to dysregulation of gastrointestinal hormones, including increased ghrelin secretion, which enhances appetite and diminishes satiety (68). These changes collectively elevate the risk of gallstone development. This suggests that night shift work and genetic susceptibility collectively elevate the risk of cholelithiasis through the mechanism of increased BMI. Similarly, night shift work and PRS for cholelithiasis were strongly correlated with abnormalities in lipid metabolism and bile acid homeostasis (29, 30, 69, 70). Therefore, night work might and genetic risk together influence the occurrence of cholelithiasis by means of alterations in biochemical markers that are closely associated with the emergence of cholelithiasis. Lastly, night shift work might eventually result in decreased gallbladder motility by suppression of melatonin production (55, 71). Some of the loci associated with the PRS of cholelithiasis may influence the development of cholelithiasis by affecting gallbladder contractility and cholestasis (31). Therefore, the combined effects of night shift work and genetic risk on gallbladder contraction might aggravate the occurrence of cholelithiasis. Based on our results, we suggest that further research should focus on whether genetic risk and environmental exposure further exacerbate disease by jointly affecting parts of the disease pathogenesis. These results highlight the need for personalized prevention strategies and suggest that future primary prevention efforts for night shift workers should consider individual genetic susceptibility.

The strength of our study lies in its prospective design, large sample size, long-term follow-up, and detailed current shift work information. Notably, this is the first cohort study to investigate relationships between night shift work and cholelithiasis risk, while also assessing the contribution of genetic factors in these relationships. This enabled us to precisely determine the effects of night shifts on groups with varying levels of susceptibility. Additionally, a series of sensitivity analyses were conducted to show the robustness of the findings. In addition, we have further clarified the roles of multiple lifestyle factors, helping to guide and improve strategy development and practice in health areas related to shift work.

Some limitations also remained in our study. First, the nature of observational research prevents both the exclusion of residual or unmeasured confounders and the inference of identified associations to causation. Further studies are warranted to confirm causation. Second, current and lifetime employment information and lifestyle data were self-reported; and hence, it is likely to be prone to some degree of classification error. However, as mentioned in previous studies (72), the misclassification would likely bias our results toward the null and thus underestimated due to the presence of potential misclassification. Third, the assessments of current and lifetime employment and lifestyle information in this study were based on baseline levels rather than being dynamic which may not accurately represent long-term status. The associations of long-term trajectories of occupational status with cholelithiasis could not be examined. Reassuringly, all participants at the time of recruitment were middle-aged or older adults whose lifestyle habits should have been well established, suggesting that their habits are likely to have remained consistent over years between recruitment and diagnosis of cholelithiasis. Future research should give priority to life course methods. Fourth, the participants recruited by the UK Biobank are not representative of the UK general population in terms of socioeconomic characteristics and lifestyle factors because of a healthy volunteer selection bias (73). Assuming participants are healthier than the UK general population, the hazard ratios (HRs) should be interpreted with caution. Nevertheless, the relative associations of risk factors with disease outcomes in the UK Biobank were tested to be generalizable and comparable to those from other representative cohort of general population (74, 75). Fifth, the study predominantly included white participants, limiting generalizability to Asian and Non-Hispanic Black study populations. Thus, whether the present findings could be generalized to other populations need to be further verified. Sixth, even though the mediation analysis has considered lifestyle factors, there are still some possible mediators unexamined, such as psychosocial factors and blood biomarkers. Future studies are needed to further explore more potential pathways linking night shift work with cholelithiasis. Seventh, this study did not assess some factors associated with the formation of gallstones, such as metabolic syndrome, type 2 diabetes, hematological disorders, and previous gastrointestinal surgeries (e.g., bypass surgery) (76–78). The absence of these factors may limit the comprehensiveness of the study’s findings. Eighth, loss to follow-up may have led to selection bias, potentially underestimating the true association between shift work and outcomes. Future studies should incorporate competing risk analyses. Lastly, even though night shift work showed association with cholelithiasis in our study, cholelithiasis remains a multifactorial disease, and night shift work, lifestyles, and genetic factors alone could not explain the trends in cholelithiasis incidence across countries, as other demographic and environmental factors are involved. However, notwithstanding these limitations, the prospective design, large sample sizes, extended follow-up, and consistency of results in sensitivity analyses partly allay concerns over reverse causality of the observed associations and reassure that our results are valid.

## Conclusions

In conclusion, night shift work, particularly with higher frequency and longer duration, was significantly associated with an increased risk of cholelithiasis. Our study also revealed that the risk of cholelithiasis associated with night shift work is exacerbated by higher genetic risk. These findings highlight the importance of reducing the burden of cholelithiasis through intervention of potential mediators.

## Data Availability

Publicly available datasets were analyzed in this study. This data can be found here: UK Biobank is an open access resource, and the study website https://www.ukbiobank.ac.uk/ has information on available data and access procedures. Data sets used for the analysis will be made available under reasonable requests.
